# Caffeine and Sports Performance: The Conflict between Caffeine Intake to Enhance Performance and Avoiding Caffeine to Ensure Sleep Quality

**DOI:** 10.1007/s40279-025-02245-y

**Published:** 2025-06-27

**Authors:** Hugo Silva, Juan Del Coso, Craig Pickering

**Affiliations:** 1 Research Center in Sports Sciences, Health Sciences and Human Development, CIDESD, University of Maia, Maia, Portugal; 2 University of Maia, Maia, Portugal; 3 Sport Sciences Research Centre, Rey Juan Carlos University, 28943 Madrid, Spain; 4 Australian Athletics, Melbourne, Australia

## Abstract

The ergogenic effects of caffeine have been reported in scientific literature over a wide spectrum of sporting activities. The current recommendation for caffeine supplementation is ingesting ~ 3–6 mg/kg about 1 h before the onset of exercise. However, some studies reporting caffeine-induced ergogenic effects during exercise have also reported increased activation and reduced sleep quality in the hours after caffeine ingestion. While most of the research on caffeine supplementation for sporting activities recommends the consumption of this stimulant to enhance performance, research focusing on athletes’ sleep quality advises against this decision, especially before competition in the evening or later. Considering that some athletes often compete in the evening or later, the general recommendation of caffeine supplementation may be modified for these athletes as acute caffeine intake in these conditions may produce undesirable side effects such as insomnia and, potentially, reduced performance in subsequent days. In this review, we examine literature on this topic to help athletes and sports practitioners solve the dilemma between the convenience of using caffeine to enhance sports performance or avoiding caffeine to ensure sleep quality. This review identifies potential solutions for this decision, keeping the focus on athletes’ well-being. Overall, the performance response to caffeine and the effect of this substance on sleep quality can vary interindividually and depend on the conditions of the exercise session (time of onset, duration, etc.). For this reason, nutritional practitioners should assess their athletes individually to resolve the conflict between caffeine ingestion and ensuring sleep quality on an individual basis, using simulated competitions with dual measurement of performance during exercise and unwanted effects in the following hours. In addition, the dose, timing, and source of caffeine supplementation can be individually adjusted to obtain performance benefits while reducing side effects for athletes ingesting caffeine before evening sporting events.

## Key Points

**Table:** 

Caffeine supplementation can enhance performance but negatively affect sleep quality, creating a potential conflict.
Considering that responses to caffeine can vary interindividually, each athlete should be carefully monitored for potential benefits and detriments.
Experimentation with specific strategies prior to competition can potentially avoid negative effects.

## Introduction

Coffee is one of the most highly consumed beverages around the world, with an estimated 2.25 billion cups of coffee consumed globally per day [1]. The reasons for this high consumption of coffee are many and varied but include coffee’s use as a social drink, habitual intake, taste, or for enhanced alertness and performance [2, 3]. In addition, research demonstrates that many individuals utilize coffee to mitigate the drawbacks of sleep deprivation [4]. Besides coffee, caffeine can also be ingested through energy drinks or soft drinks, tea, tablets, chewing gums, gels, and even nasal and mouth aerosol sprays [5, 6]. Caffeine’s physical performance benefits have been widely investigated and appear to be mostly driven via caffeine’s (1,3,7-trimethylxanthine) blockade of adenosine receptors [7–10] that leads to the inhibition of the negative neuromodulation effects of adenosine. As the performance benefit of caffeine is well established [11, 12], it is unsurprising that both coffee consumption and caffeine supplementation are of interest within the sports community.

When exploring the ergogenic effects of caffeine, previous research highlighted that caffeine supplementation enhances the volume of work carried out [13, 14], reduces glycogen utilization [15] during prolonged exercise, and increases muscle tension with or without fatigue [16]. In addition, caffeine supplementation also improves muscle strength and power [17], improves time trial performances during cycling [18] and running activities [19], and shows ergogenic effects in combat [20] and team sport athletes [21]. Both male and female athletes benefit from caffeine supplementation with similar performance magnitudes [22]. Caffeine’s performance-enhancing effects are so potent that its use was previously banned in competition from 1984 to 2004 [23], before being removed from the substance doping list in 2004 by the World Anti-Doping Agency (WADA) [23]. Studies measuring urine caffeine concentration before and after removal of caffeine from the doping list reflect that in both cases a high proportion of athletes had caffeine concentrations compatible with low-to-moderate ingestions of sources of caffeine. This indicates that the prior ban on caffeine was not effective in limiting its use in sports and that caffeine is a substance widely ingested in sports (around 75% of athletes present caffeine in their urine samples collected after exercise) [24]. Nevertheless, caffeine is still being monitored by WADA because, despite its performance benefits, it is not an innocuous substance [25].

Although no longer banned, caffeine consumption may elicit side effects, including nervousness, irritability, diuresis, gastrointestinal problems, and insomnia [26–32]. These side effects may be driven by the amount of substance ingested, making the dose of caffeine (often reported in terms of mg per kg of athletes’ body mass, i.e., mg/kg) of interest. Previous research has highlighted the wide range of caffeine content within decaffeinated coffee (ranging from 0 to 13.9 mg), espressos (58.1–185 mg), and specialty coffees (143.4–259.3 mg) [33, 34]. Similar research highlighted the variation present in pre-workout supplements when compared with the advertised dose [35]. This variation present within common sources of caffeine makes it difficult to accurately quantify the total amount ingested before a sporting activity. While the suggested ergogenic dose of caffeine ranges from 3 to 6 mg/kg [36–38], higher doses may increase the risk of side effects without producing a further performance benefit than the traditional 3–6 mg/kg range [39]. The time of caffeine ingestion may also be a confounding factor. While caffeine typically peaks within saliva and plasma around 60 min post-ingestion [40, 41], it tends to be consumed close to competition [37, 38, 42]. All of the above raises a potentially problematic issue in the sporting setting; if competition is in the evening or later, pre-competition caffeine ingestion may be ergogenic but may also disrupt sleep [43–45], ultimately having a negative impact on crucial aspects for athletes such as recovery, well-being, and subsequent performance [46–48].

It is well established that sleep is fundamental to human health [49–57]. Accordingly, athletes require sufficient sleep duration and quality to support their performance. As an example, disturbances of sleep are suggestive of overreaching/overtraining [58] and appear to increase the risk of injury [59]. In addition, adequate sleep improves players’ recovery and performance [48, 60, 61]. Given caffeine’s wide use, which is, in part, driven by its ability to increase alertness and reduce drowsiness, a common recommendation to improve sleep quality is to avoid caffeine consumption, especially near bedtime [32, 44, 45, 62–64]. As such, there is an apparent conflict between caffeine’s ergogenic effects and its negative effects on sleep quality and quantity, with practitioners needing to prioritize strategies to improve athletes’ performance without compromising their well-being. Considering that many athletes compete in the evening or later, or engage in multiday sports competition, the general recommendation of caffeine supplementation for exercise could be adapted for these athletes. This is because acute caffeine intake following the general recommendations explained above may produce undesirable side effects such as insomnia and ultimately worse performance in the next competitive situation. Therefore, this review intends to summarize the benefits of caffeine ingestion and adequate sleep on sports performance, while evaluating if both strategies can coexist.

To achieve this, a comprehensive literature review was conducted to examine the effects of caffeine intake and sleep quality on sports performance. The review followed a three-step approach. First, systematic searches for original studies and review articles on this topic were performed using PubMed, the Web of Science, and SPORTDiscus using a mix of Medical Subject Headings (MeSH) and free-text words for key concepts related to the effect of caffeine on sleep: concept 1 (caffeine OR coffee OR energy drinks) AND concept 2 (sleep OR sleep quality OR insomnia) AND concept 3 (athlete OR sport OR exercise). Expert recommendations and backward citation searches were utilized to supplement database retrieval. Second, all titles from the search were downloaded to a Microsoft Excel spreadsheet and manual cross-referencing was performed to identify duplicates. Third, titles and abstracts were then screened to identify studies on the topic. We only incorporated studies in which the effect of the ingestion of caffeine on athletes’ sleep parameters was analyzed or reviewed. To maintain a specific focus on sports performance, studies involving amateur or recreational athletes, children, and animal studies were excluded. In addition, studies that did not directly investigate sports performance within the context of caffeine and sleep were excluded, except for those providing essential insights into key concepts relevant to this review.

## Caffeine

As previously discussed, caffeine exerts its ergogenic effects through several proposed mechanisms. After ingestion, caffeine is rapidly absorbed, leading to an increase in caffeine concentration in plasma (within 1 h) [40]. Among other actions within the human body, caffeine may induce an increase in calcium release in the muscle fiber and an attenuation of circulating potassium [40], which increases the potential of the resting membrane and contributes to enhanced muscle contraction [65]. However, the most plausible mechanism behind caffeine’s ergogenicity across a wide variety of exercise situations is its role in the central nervous system. Caffeine is a competitive adenosine receptor antagonist [7], specifically for receptors A_1_ and A_2A_ [10]. Without this blocking action, extracellular concentrations of adenosine can increase with intense exercise, promoting fatigue [66–68]. As adenosine exerts some “fatiguing” effects during exercise through the inhibition of the release of excitatory neurotransmitters, the blockage of its receptors after caffeine intake produces just the contrary effect—a higher release of excitatory neurotransmitters such as dopamine and noradrenaline [69]. By increasing dopamine levels [70], a substance known for its stimulant effects [71–74], free fatty acid mobilization may occur, potentially enhancing fuel delivery to muscles [65]. This mechanism may be beneficial in exercise situations where fat serves as an important energy substrate, highlighting its relevance primarily for low-to-moderate intensity aerobic exercise. However, caffeine’s primary mechanism for enhancing exercise capacity is central stimulation, as the local effects are typically observed at doses that exceed those commonly used for ergogenic purposes.

In sports, the stimulant effect of caffeine on the central nervous system affects athletes’ vitality (increasing) and perceived exertion (decreasing) after consuming caffeine [75]. Therefore, the intake of caffeine about 1 h before exercise allows the blockade of adenosine receptors by caffeine that in turn impedes the fatiguing effects of adenosine during exercise, favoring higher exercise intensity or lower perceived fatigue or pain for clamped-intensity exercise.

### The Ergogenic Effects of Caffeine

A plethora of studies report improvements in sports performance after caffeine ingestion. For example, when compared with a placebo, caffeine ingestion attenuated decrements in repeated sprint performance and reactive agility times of team sport athletes [76]; improved passing accuracy, jump performance, aerobic endurance (increased time to fatigue during a treadmill test) of soccer players [77, 78]; improved sprint, power, and accuracy tasks of rugby players [79]; improved ball-throwing velocity, jump, and sprint performances of female handball players [80]; improved jump performance in volleyball players [81]; increased offensive actions during combat sports [82]; and improved performance during cycling [83] and running [84] endurance activities. In addition, athletes reported lower ratings of perceived exertion (RPE) during submaximal aerobic [85] and anaerobic (Wingate test) [86] activities while increasing the ventilatory threshold [87] and oxygen uptake [88] and reducing the heart rate [89] during submaximal activities. Finally, performance benefits are also expanded to cognitive functions, improving attention, mood, reaction time, and memory [90]. To assess the benefits of caffeine ingestion, two important factors should be highlighted: the amount of caffeine ingested and the time of ingestion. In trials, the amount of caffeine ingested varied from 2.1 mg/kg to 6 mg/kg and was typically ingested ≤ 60 min (ranging from 5 to 60 min) prior to physical tests or activities [76–81, 85, 86]. Interestingly, the recommendations of the International Society of Sports Nutrition remained the same (ingestion of 3–6 mg/kg of caffeine ≤ 60 min prior to the activity) in two publications 13 years apart [37, 91]. Here, we should highlight the different caffeine forms of consumption. For example, providing caffeine supplementation in a gum format increases the rate of absorption when compared with a capsule format [92]. Importantly, the ergogenic effect appears to be more dependent on providing the correct amount of caffeine with an individual approach rather than the source of caffeine ingestion [6, 93]. Exceptions could exist regarding the effectiveness of caffeine supplementation in aerosols and mouth rinses formats, owing to the scarcity of research on these formats [6, 94].

However, it is important to recognize that even if athletes show benefits in some specific tests (such as maximal oxygen consumption (*V*O_2max_) or jump tests), those benefits do not necessarily translate to performance enhancement during competition (ecological benefit). For instance, previous research has not reported reliably clear performance benefits during soccer, rugby, ice hockey, and basketball matches or simulated matches [95–98], while the opposite was found during futsal simulated matches [99]. Nevertheless, one meta-analysis [21] identified caffeine benefits in team sport performances with players covering greater running and sprint distances (mean difference with 95% confidence intervals [95% CI] of 0.41 [0.20, 0.62] and 0.36 [0.12, 0.59], respectively) and number of sprints (0.44 [0.18, 0.69]). This analysis was based on six studies, with most protocols providing 3 mg/kg of caffeine 60 min before the task [100–104], while one experimental group consumed 6 mg/kg [95]. Interestingly, the higher dose did not result in clear advantages to match-play performance. Still, the need for further research on this topic was highlighted in two recent reviews [38, 105]. Therefore, assessing the potential benefits of caffeine intake in team sports is challenging owing to the influence of various factors, and these findings should be interpreted with caution.

### Variability of the Ergogenic Effects of Caffeine

#### Genetic Heterogeneity

In addition, there is some heterogeneity in response to caffeine ingestion [93], resulting in differences in both ergogenic and ergolytic effects based on a variety of factors. Among these factors, variations in two genes (*CYP1A2* and *ADORA2A*) appear to influence the individual response to caffeine as they encode proteins critical for caffeine metabolism and its mechanism of action, respectively. The *CYP1A2* gene codifies the CYP1A2 enzyme that is responsible for 90% of caffeine metabolism. A single nucleotide polymorphism in the *CYP1A2* gene, known as c.-163A > C (rs762551), has been proposed as a potential explanation for the interindividual variability in response to caffeine as this genetic variant modifies the activity of the CYP1A2 enzyme. Specifically, individuals who are homozygous for the AA genotype at this genetic variant exhibit faster capacity to metabolize caffeine into paraxanthine and other dimethylxanthines compared with those with the CA or CC genotypes [106]. According to this notion, AA homozygotes may metabolize caffeine into paraxanthine more rapidly than CA and CC individuals, leading to a faster clearance of caffeine from the bloodstream [107]. Consequently, this accelerated metabolism of caffeine in athletes with the *CYP1A2* AA genotype may render them less sensitive to its effects, as caffeine is cleared more rapidly. This faster clearance could diminish the ergogenic impact of a given dose compared with athletes with the AC or CC genotypes [108]. However, an alternative hypothesis has been proposed, suggesting that AA genotype athletes may be more sensitive to caffeine, as some studies have reported a greater ergogenic response in AA individuals compared with C-allele carriers [109]. Despite the well-established influence of the *CYP1A2* c.-163A > C variant on the rate of caffeine metabolism, current evidence suggests that this genetic polymorphism may have little impact on caffeine-induced ergogenic effects [110, 111] and sleep disturbances [112, 113], suggesting that differences in caffeine metabolism speed may not be a primary factor in modulating the acute response to caffeine intake. This could be because caffeine’s primary metabolite, paraxanthine, also has stimulant effects, potentially contributing to exercise performance and sleep disruption regardless of metabolism rate [114].

The *ADORA2A* gene codifies the A_2A_ receptor, which plays a major role in the binding process between caffeine and adenosine receptors, as explained previously [115]. The 1976C > T (rs5751876) polymorphism in the *ADORA2A* gene has also been proposed as a modifier for the response to acute caffeine intake. Specifically, the *ADORA2A* gene has been used to categorize individuals as “high” (TT genotype) or “low” (CC/CT genotype or C-allele carriers) responders to caffeine [116]. Similar to the previous genetic variant, current evidence indicates that individuals with different ADORA2A genotypes exhibit comparable responses to caffeine in terms of enhanced exercise performance [110, 111, 117]. However, it seems that, in this case, the *ADORA2A* polymorphism may affect the effect of caffeine on sleep, as C-allele individuals are more prone to sleep disturbances after caffeine intake than T-allele carriers [112, 118]. In summary, on the basis of these two genes, previous research has classified individuals as “slow” caffeine metabolizers (*CYP1A2* C-allele carriers) and “fast” (*CYP1A2* AA genotype) caffeine metabolizers and into “low” (*ADORA2A* TT genotype) or “fast” metabolizers (*ADORA2A* C-allele carriers) and as having “high” sensitivity to caffeine (*ADORA2A* TT genotype) or “low” sensitivity (*ADORA2A* C-allele carriers) [119]. However, in the context of exercise, various studies have reported similar ergogenic effects of caffeine, despite interindividual differences in these genotypes [111, 120]. All of this suggests that interindividual variability in response to caffeine may be somewhat influenced by genetics, among other factors. However, more robust studies with larger sample sizes and the inclusion of additional candidate genes are needed to better understand how genetics impact caffeine’s effects on exercise performance and sleep.

#### Habitual Consumption of Caffeine

Given that caffeine is habitually consumed in daily life, athletes who perceive ergogenic effects may continue using it prior to training in an effort to maintain or enhance performance. For this reason, research has explored whether the habitual use of caffeine diminishes caffeine’s ergogenic benefits over time [88, 121]. This phenomenon is known as tolerance to the effects of caffeine and potentially occurs when the body adapts to regular caffeine consumption, leading to a reduced physiological and performance-enhancing response over time. Tolerance to caffeine was initially explored in animals, with an increase in adenosine receptors as an adaptation to chronic caffeine consumption [122–125]*.* More recently, physiological and exercise performance responses compatible with tolerance to caffeine have also been reported in humans [126–128]. In practice, this means that the performance benefits of acute caffeine intake may progressively decrease with chronic caffeine consumption [88, 121].

Importantly, contrasting findings have also been reported regarding the existence of tolerance to caffeine in an exercise context [129]. For instance, several cross-sectional studies comparing the effects of acute caffeine intake in naïve and habitual caffeine consumers have found that both groups experience similar performance benefits from acute doses of 3–6 mg/kg [130, 131]. In addition, a recent systematic review that summarized cross-sectional studies exploring the influence of habitual consumption on the acute exercise response to caffeine supplementation revealed that habitual caffeine consumption did not influence the acute ergogenic effect of caffeine [129]. However, crossover studies involving participants who underwent controlled habituation to caffeine through daily intake for up to 28 days have shown that caffeine’s ergogenic effects are strongest on the first day of consumption, with a diminished response observed as habituation progressed [87, 88, 121]. Interestingly, crossover studies have shown that while caffeine’s ergogenic effects progressively diminish after 20–28 days of chronic intake, it remains effective in enhancing performance after this period. Overall, findings from both cross-sectional and crossover studies suggest that tolerance to caffeine develops gradually and at a slow pace. This means that even athletes with high habitual caffeine intake can still experience significant performance benefits from acute caffeine supplementation. In this regard, the relationship between habitual caffeine consumption and tolerance to its effects on sleep disturbances is complex. Some studies suggest that regular caffeine users may develop a tolerance to caffeine-induced sleep-disrupting effects [132, 133] while others indicate that habitual consumption does not eliminate these disturbances. Further research is needed to clarify how habitual caffeine use influences sleep disturbances, especially in athletes. In addition, more research is needed to better understand the extent and mechanisms of caffeine tolerance, particularly about its ergogenic effects and potential impact on sleep, to help athletes optimize their caffeine consumption for performance benefits while minimizing adverse effects.

Following the research on caffeine habituation, two main strategies have been explored to counteract a potential decline in responsiveness over time: caffeine withdrawal or re-sensitization, or simply increasing the amount of caffeine ingested. The first strategy has been widely contested owing to reported adverse effects, including headache, irritability, increased fatigue, decreased alertness, difficulty concentrating, nervousness, depressed mood, muscle pain, and decreased energy [39, 134]. In addition, performance benefits can be absent [135, 136] or, if registered, attributed to a reversal of the adverse effects [137, 138]. The effectiveness of the second strategy (i.e., increasing the amount of caffeine ingested) is also unclear. Moreover, increasing caffeine consumption above 6 mg/kg appears to be ineffective in providing additional benefits [129]. Therefore, while increasing the amount of caffeine can mitigate the attenuation of caffeine’s ergogenic effects [93], one should keep in mind the different individual responses to caffeine and a potential increase in the side effects (such as sleep deprivation) with higher doses [39, 45, 93, 139].

### Caffeine’s Performance-Enhancing Effects May Potentially Affect Sleep

While the stimulating effects of caffeine can be beneficial during exercise training or competition, they cannot be immediately switched off once the exercise is completed. The prolonged presence of caffeine in the system may delay the body’s transition into a recovery state, potentially impairing post-exercise relaxation, reducing sleep quality, and disrupting overall recovery processes. Since the half-life of caffeine is relatively short (i.e., the elimination from the body of half of the ingested caffeine ranges from 2.5 to 10 h after ingestion) [40], consuming caffeine before a morning competition or training session can enhance performance without compromising nighttime sleep. This is because enough time will have passed for a substantial portion of caffeine to be metabolized and excreted before bedtime. Still, taking into account the prolonged stimulant effect of caffeine is relevant for athletes who train or compete in the evening, as residual caffeine stimulation may interfere with sleep onset, duration, and quality, ultimately affecting subsequent performance and recovery.

## Sleep

Sleep has several functions, playing an important role in emotional regulation, homeostatic restoration, immune control, memory processing, mood regulation, thermoregulation, and tissue repair [49, 51–53, 55, 140]. Therefore, it is not surprising that sleep deprivation negatively affects overall health, including raising proinflammatory markers, which contribute to the development of illness [141]. Although exercise assists in avoiding sleep disorders [142], such as relieving insomnia [143], athletes generally have less quality sleep than they need [60, 144–147] and less than non-athletes [148]. This poor sleep quality places athletes at high injury risk [58, 59, 146] and decreases performance [146, 149]. It is important to highlight how sleep quality is assessed. The use of polysomnography and actigraphy is uncommon in sports owing to high costs and the need for experts to correctly assess the retrieved data [150, 151]. Consequently, practitioners usually select sleep questionnaires such as the Pittsburgh Sleep Quality Index and the Athlete Sleep Behavior Questionnaire [150, 151]. However, these questionnaires are subject to participant bias, where an individual can have an incorrect perception of sleep quality.

### Sport Impairing Sleep Quality

Although training load can impact sleep quality [152–154], schedules and routines have a major role in sleep. For example, athletes can suffer more sleep constraints owing to early morning practices or multiple sessions during the day [145, 154, 155]. In addition, athletes must sometimes compete during the night, which delays the bedtime owing to the late time of the event, subsequent post-competition arousal, post-competition socializing, and media interactions [156–164]. The increase in arousal levels, due to high-intensity activities [165], can delay sleep initiation and disrupt sleep, caused by an overactivation of the sensory processing systems [166, 167]. Increasingly, by competing at night, athletes are exposed to bright light [168] that impacts the human circadian rhythm and the concentration of melatonin in plasma [169].

## Caffeine versus Sleep

### Does Caffeine Impair Sleep Quality and Quantity?

**Fig. 1:**
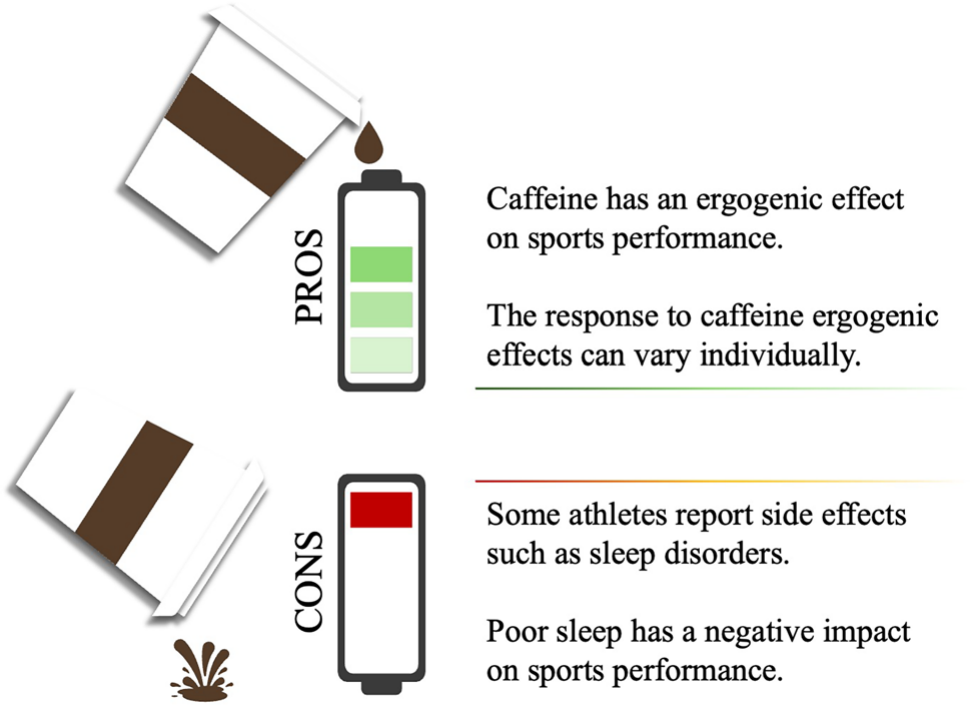
Pros and cons of caffeine supplementation in sports performance: a potential conflict

Caffeine is often used to increase alertness, and, in particular, during periods of acute or chronic fatigue associated with sleep inadequacy [170]. As it is used in this way, it is logical to assume that caffeine, when consumed later in the day, may affect sleep latency, quality, and duration. Drake and colleagues [44] reported that 400 mg of caffeine consumed 6 h prior to bedtime had a significant negative effect on sleep quality compared with placebo. Similar results have been reported in professional sports. For example, Dunican and colleagues [171] investigated the influence of caffeine ingestion on subsequent sleep following an evening Super Rugby game, reporting that changes in salivary caffeine concentrations were moderately related to increases in sleep latency and decreases in sleep efficiency. Similar findings have been reported in other athlete cohorts [172, 173]. Given the importance of post-competition sleep, and sleep in general, on athlete recovery and subsequent performance, these results highlight a potential conflict—while caffeine is performance-enhancing, could its use in evening or afternoon competition have subsequent negative effects (Fig. 1)? Increasingly, the presence of anxiety symptoms appears to increase caffeine consumption [174], increasing the detrimental effect of caffeine on sleep. This is of particular concern, as some athletes are prone to anxiety due to injuries, career dissatisfaction, performances, and adverse life events [175]. That is, some athletes may be tempted to consume more caffeine to cope with anxiety, or to compensate for a poor night’s sleep and to ensure functioning during daytime activities [176].

### Do We Have to Choose Between Caffeine and Sleep?

As highlighted within this paper, studies that discuss caffeine’s ergogenic effects recommend its consumption ≤ 60 min prior to the activity, while studies that discuss the benefit of having quality sleep recommend avoiding caffeine near bedtime. As such, a conflict clearly exists. The first step toward a solution is acknowledging that consuming caffeine, even if 5–6 h before bedtime, will likely disrupt sleep [44, 172]. Secondly, the caffeine–sleep relationship can also be seen inversely, i.e., caffeine consumption can be a strategy to compensate for poor sleep [176, 177]. For example, one study [178] compared the effects of a 20-min nap, 5 mg/kg ingestion of caffeine, and combining both strategies as counterstrategies to partial sleep deprivation, with the combined strategy presenting higher performance benefits. This is important because athletes can have poor sleep quality owing to several reasons (such as competing at late schedules or training early in the morning) and rely on caffeine to counteract sleep deprivation while affecting subsequent sleep quality.

As little as one cup of coffee can have a negative effect on sleep [43, 45], even when consumed well before an individual’s normal bedtime. The half-life of caffeine appears to vary widely in humans, with studies reporting intervals from 4 to 6 h [2], 2.5 to 10 h [40], and 2 to 12 h [179]. Increasingly, interindividual variations of caffeine’s effects, due to certain genetic characteristics or other factors such as age or caffeine habituation, have been studied, which may affect the speed at which caffeine is metabolized [39, 93]. As such, we need to ensure that guidelines around sleep and caffeine use are individualized. Individuals can respond to a low [180] (or even no (placebo)) [181] amount of caffeine. Similarly, an athlete can report poor sleep due to nervousness prior to the competition [182], and ingestion of caffeine can exacerbate his or her anxiety [2]. Therefore, athletes should experiment with caffeine dose and time of ingestion prior to competition [39, 93] while monitoring sleep quality with the help of the coaching staff [60, 151]. Before this individual analysis, athletes should refer to sleep hygiene guidelines to avoid compromising health, well-being, and performance.

### Strategies to Improve Sleep Quality

Unfortunately, to cope with sleep disturbances, some athletes resort to pharmaceuticals with some risk of undesirable effects, and even addiction [183, 184]. To avoid addiction and negative impact on performance, sports psychiatrists often recommend the use of melatonin to help athletes with insomnia [185]. In addition, several studies have provided several safe and simple strategies to improve sleep quality. Although practitioners understand the importance of sleep hygiene [186], implementing good practices is fundamental to obtaining the desired outcome [187]. Moreover, practitioners should establish how sleep quality will be assessed. Importantly, because technological developments can facilitate sleep assessment, athletes can increase the risk of developing “orthosomnia”—the obsessive pursuit of perfect sleep [188–190]. As an alternative to technological devices, validated questionnaires are simple to use and inexpensive, delegating the monitoring task to practitioners [60, 151, 191]. After assessing and identifying sleep disorders, practitioners should keep in mind the importance of referring those athletes to clinical specialists [60, 61]. Nevertheless, several strategies can be implemented to improve athlete’s sleep quality: exposure to natural light in the morning [60, 61, 168, 192]; implementing short naps [48, 60, 61, 168, 193]; avoiding excessive fluid intake before bedtime [140, 192]; preparing a cool, dark, and quiet room [48, 60, 168, 192]; avoiding the use of electronic devices before bedtime [168, 192]; having a regular a bedtime routine/schedule [61, 168, 192]; and avoiding the consumption of caffeine after lunch or near bedtime [60, 61, 140, 192].

### Practical Applications

**Fig. 2:**
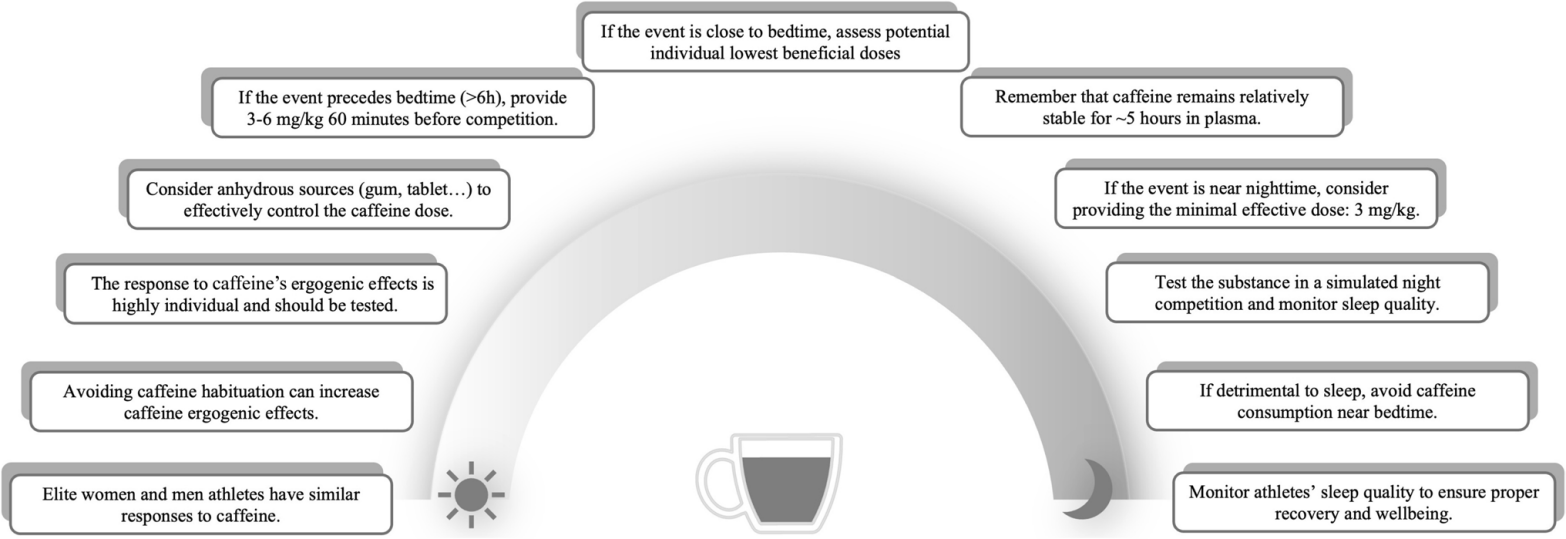
Practical recommendations to exploit caffeine ergogenic effects without compromising sleep quality

Assessing the potential benefits of caffeine on sports performance is crucial for understanding how it can enhance athletic outcomes. However, it is equally important to consider the potential constraints caffeine may place on sleep quality, as disrupted sleep can ultimately undermine its ergogenic effects. These factors should be carefully weighed when exploring specific strategies for caffeine use. Specifically, practitioners can apply different strategies such as: (1) considering the individual responses to caffeine, regardless of the similarities between female and male athletes; (2) avoiding regular caffeine use may help prevent tolerance, thereby enhancing its performance-boosting effects even at lower doses; (3) testing individual responses to caffeine, preferentially in a simulated competition; (4) experimenting with different sources of caffeine, such as capsule, tablet, or gum to better control the dose; (5) following the general recommendations of dosage (3–6 mg/kg) if the event occurs several hours before bedtime while remembering that caffeine remains relatively stable for 5 h in plasma; (6) experimenting with the individual lowest beneficial dose if the sporting activity occurs a few hours before bedtime; and (7) monitoring sleep to ensure effective recovery and to assess potential side effects of caffeine (Fig. 2).

## Conclusions

Both caffeine ingestion and sleep impairments have been widely studied in scientific research. However, studies that investigated the performance-enhancing effects of caffeine recommend the ingestion of caffeine 60 min before the specific task, while studies that investigated potential issues in sleep advised against caffeine ingestion close to bedtime. Therefore, a conflict exists regarding night events. Practitioners and athletes should consider the importance of individuality, which means that each athlete can face different challenges. Specifically, some athletes may benefit from low doses of caffeine, while others may require higher doses or even avoid caffeine completely owing to side effects. Similarly, one athlete may struggle in coping with anxiety near competition, impairing his or her sleep, while another athlete can fall asleep easily, even if consuming caffeine near bedtime. As so, considering that poor sleep quality impacts both the performance and health of athletes, sleep hygiene guidelines should be considered while experimenting with potential individual caffeine benefits and side effects. Practical recommendations were provided to strategize the consumption of caffeine while avoiding compromising sleep quality.
